# Developing an alcohol strategy for the Northwest Territories: Evaluating global research evidence against rural and remote realities

**DOI:** 10.17269/s41997-024-00899-1

**Published:** 2024-06-27

**Authors:** Bryany Denning, Paul Andrew, Pertice Moffitt, Barbara Broers

**Affiliations:** 1 Faculté de Médecine, Université de Genève, Geneva, Switzerland; 2 Department of Health and Social Services, GNWT, Yellowknife, NT Canada; 3 Yellowknife, NT Canada; 4 Aurora Research Institute, Yellowknife, NT Canada

**Keywords:** Alcohol policy, Harm reduction, Northwest Territories, Canada, Alcohol use prevention, Youth, Indigenous, Politique sur l’alcool, réduction des méfaits, Territoires du Nord-Ouest, Canada, prévention de la consommation d’alcool, jeunes, Autochtones

## Abstract

**Objectives:**

This paper outlines the engagement process that was used to develop the Northwest Territories Alcohol Strategy, based on a recommendation by the developers of the Canadian Alcohol Policy Evaluation report, and how this informed the final actions in the strategy.

**Methods:**

A literature review, four targeted engagement activities, and iterative validation by advisory groups and community and Indigenous leadership were used to evaluate, modify, or reject the original recommendations and develop the final actions that were included in the NWT Alcohol Strategy.

**Results:**

There are fourteen original CAPE recommendations, four of which had already been implemented in the Northwest Territories before the development of the strategy. On completion of the process, four recommendations had already been implemented in the NWT. Two recommendations were included in the strategy without changes, two were adapted for use in the strategy, and six were not included. One stand-alone alcohol policy measure was created and included.

**Conclusion:**

Alcohol strategies are dependent on a variety of contextual factors. Developers need to take into consideration the unique geography, political climate, and cultural context of the region for which they are being developed, in order to produce a strategy that is applicable, acceptable, and feasible at the community level.

## Résumé

**Objectifs:**

Cet article présente la méthodologie de projet utilisée pour élaborer la Stratégie sur l’alcool des Territoires du Nord-Ouest (TNO), basée sur un ensemble de recommandations formulées par les auteurs du rapport d’évaluation des politiques canadiennes sur l’alcool (CAPE). Il présente également les orientations et actions finales de la stratégie.

**Méthodes:**

Une analyse documentaire, un dispositif de concertation multi-canal, ainsi qu’une validation itérative par des groupes consultatifs, des représentants des populations locales et autochtones ont été utilisées pour évaluer, modifier ou rejeter les recommandations originales et élaborer les actions finales à inclure dans la Stratégie sur l’alcool des TNO.

**Résultats:**

Le CAPE comprend quatorze recommandations d’origine, dont quatre étaient déjà en vigueur dans les TNO avant l’élaboration de la stratégie. À l’issue du processus, quatre recommandations avaient déjà été mises en œuvre dans les TNO. Deux recommandations ont été incluses dans la stratégie sans modifications, deux ont été adaptées pour être mises en œuvre dans la stratégie, et six n’ont pas été incluses. En outre, une politique indépendante de quantification a été créée et incluse.

**Conclusion:**

Les stratégies en matière d’alcool dépendent de divers facteurs contextuels. Les concepteurs doivent tenir compte de la singularité géographique, du climat politique et du contexte culturel de la région pour laquelle elles sont élaborées, afin de rendre la stratégie applicable, acceptable et réalisable pour la population.

## Introduction

Over three quarters of Canadians report the consumption of at least one drink in the past year (Statistics Canada, 2021). Canadians tend to be supportive of alcohol policies that are less effective at reducing alcohol use, such as educational campaigns, and are more resistant to policies that put limits on availability and outlet density or increase alcohol prices, which demonstrate more impact (Giesbrecht et al., 2019; van der Maas et al., 2020). These attitudes persist despite experiences of harm associated with alcohol; 21% of Canadians surveyed in 2019 reported experiencing at least one type of alcohol-related harm in the past year (Statistics Canada, 2021).

Awareness of the risks associated with alcohol use is growing in Canada, with recent guidelines advising that Canadians drink no more than two drinks^1^ per week if they wish to avoid increased risk of alcohol-related harm (Paradis et al., 2023). Alcohol consumption is causally linked to oral cavity, pharynx, larynx, oesophagus, colon, rectum, liver, and breast cancers (Boffetta and Hashibe, 2006), as well as tuberculosis, diabetes, heart disease, stroke, pneumonia, and cirrhosis of the liver (Rehm et al., 2010). Alcohol is a known teratogen (Ernhart et al., 1987), and alcohol consumption increases the risk of both unintentional and intentional injuries (World Health Organization, 2021). In Canada, the societal costs of harm associated with alcohol use outpace government revenues generated from the sale and distribution of alcohol by approximately $3.7 billion per year (Sherk, 2020).

Alcohol use impacts the residents of the Northwest Territories (NWT) in ways that are measurably more severe than in the southern Canadian provinces. The NWT has the highest rate of alcohol-related hospitalizations in Canada, with 1759 hospitalizations per 100,000 population in 2019–2020, more than 6.8 times the national average (Canadian Institute for Health Information, 2023). The 2018 NWT Tobacco, Alcohol and Drug Survey found that nearly 40% of drinkers in the NWT typically consume five or more drinks per drinking occasion (NWT Bureau of Statistics, 2018). The survey also revealed high rates of harm related to alcohol, with 20% of respondents reporting that alcohol had had harmful effects on their home life or marriage, 23% reporting harmful effects on their physical health, 20% reporting harmful effects on friendships or other relationships, and 15% reporting harmful effects on educational or employment opportunities. Alcohol use has resulted in legal problems for 10% of the population, financial problems for 10%, and housing problems for 4% (NWT Bureau of Statistics, 2018).

The rates of alcohol use and alcohol-related harm in the NWT are particularly concerning considering the limited number of alcohol sales outlets, and existing liquor restrictions. Under the NWT Liquor Act, a community may, via a community plebiscite, restrict or prohibit alcohol possession and sale, or remove existing restrictions (SNWT 2007, c15, n.d.). Out of 33 communities, ten have restrictions on the type and amount of alcohol that a person may possess and/or transport within the community, and six prohibit alcohol possession or consumption within a given radius of the community. There are six communities in the NWT that have liquor stores, and one that permits off-sales; five of these communities do not have limits on purchase or possession amounts, though there is a limit of six 375 mL bottles (locally referred to as a “mickey”) per person across the NWT that is intended to reduce bootlegging (illegal resale) (Government of the Northwest Territories, 2021a). All residents of restricted or unrestricted communities who do not have a liquor store have to order liquor by mail from a store in their region (The Northwest Territories Liquor and Cannabis Commission, 2021).

### Rationale for an alcohol strategy

In 2019, the Canadian Institute for Substance Use Research (CISUR) released the second iteration of the Canadian Alcohol Policy Evaluation (CAPE), including a report card scoring the alcohol-related policies of each province and territory (Giesbrecht et al., 2013). In the report, the NWT received a failing grade in nine of eleven policy domains. One recommendation that was included in the report was that the NWT create an “alcohol-specific, government-endorsed strategy,” to be updated every 5 years (Chow et al., 2019).

When researchers responsible for the CAPE report presented the recommendations for the NWT to the Northwest Territories Liquor Commission (now the Northwest Territories Liquor and Cannabis Commission), government leadership questioned the applicability of some of the recommendations to the NWT, due to the high prices of goods, small jurisdictional capacity, and unique geography and population distribution in the territory. While recommendations were provided in the CAPE report that represent best practices on a global scale, questions were raised around the applicability of policy levers such as pricing and physical availability, in an area where the lack of retail outlets leads to exorbitant prices being set by bootleggers, or impaired driving countermeasures, in a territory where there are few roads, vast lands, and limited enforcement capacity. As well, alcohol-related harms disproportionately impact the Indigenous population (Giffort, 2009), as the ongoing impact of colonization and the legacy of the residential school system left community members few resources outside of substance use to cope with the resulting personal and intergenerational trauma. These same factors also contribute to high rates of interpersonal violence, suicide, and incarceration (Moffitt et al., 2013), exacerbated by increased alcohol use and social isolation during the COVID-19 pandemic. The underpinnings of intergenerational trauma that influence substance use in Indigenous communities complicate the context within which typical policy levers are implemented. Policies that use pricing, advertising, and health messaging to change alcohol use behaviours need to be looked at through a lens that considers the context-specific challenges. Based on these questions, it was clear that both research and community engagement would be required to determine appropriate actions to reduce alcohol-related harms in the NWT. The aim of this article is to describe and critically analyze the engagement process that led to the formulation of an alcohol strategy for the NWT.

## Methods

### Design

**Fig. 1:**
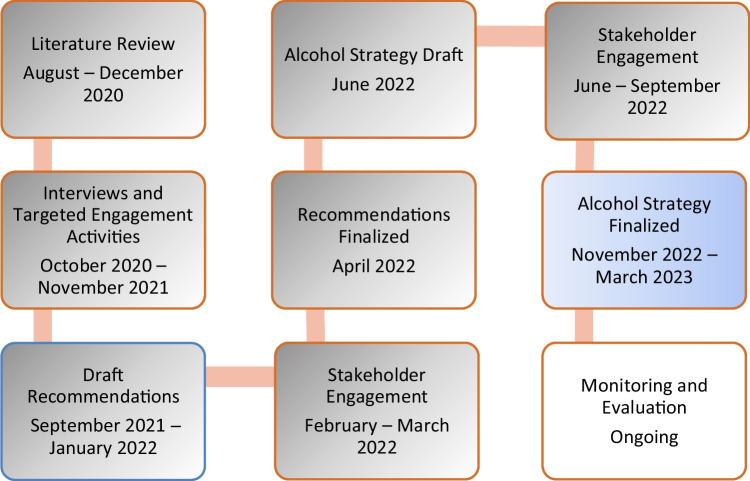
Alcohol strategy development process and timeline

The development of the NWT Alcohol Strategy was centred around the awareness that the strategy needed to include actions that were relevant to community members living in all regions of the NWT. To accomplish this, staff at the Department of Health and Social Services needed to use methods that ensured that Indigenous perspectives were centred in this process, and community members from across the north had multiple opportunities to not only provide their views but also review and revise our interpretations of what they were telling us. At the outset of the process, the senior advisor responsible for this project drafted an approach and timeline, with updates to the timeline occurring throughout the project (Figure 1). The senior advisor conducted a literature review to examine the applicability of the CAPE report recommendations to the context of the NWT, with two main aims. The first was to understand what elements of the research settings were comparable, and by extension, whether the conclusions could be generalized to the NWT. The second was to review studies on alcohol policy best practices, and other responses to alcohol-related harm, from jurisdictions that were demographically like the NWT, with a focus on those conducted in Indigenous communities, with northern populations, or in rural and remote locations.

In addition, the senior advisor did a review of past NWT reports and recommendations documents on alcohol, substance use, mental health, and addictions, and prepared a summary of alcohol strategies from other jurisdictions, particularly those developed by Indigenous populations. Foundational to this work was a review of Healing Voices, the report of the Minister’s Forum on Community Wellness and Addiction, an initiative that held discussions throughout the territory in 2012 and 2013 and summarized its findings into 67 recommendations (The Minister’s Forum on Addictions and Community Wellness, 2013). Literature on alcohol treatment modalities, including pharmacological approaches to treatment, best practices in prevention, novel initiatives, and emerging knowledge on alcohol and substance use was reviewed, as were past engagement reports with youth and other key demographics. The results of the literature review were included in a discussion paper that, combined with the target engagement activity, was used to develop preliminary recommendations.

Unstructured interviews (*n* = 16) were conducted with a wide variety of stakeholders to inform the development of alcohol strategy recommendations, and comments and insights were recorded via written or typed interview notes. Interviewees included members of the Minister’s Forum, service providers who care for people with alcohol use disorders, medical professionals, and government staff who administer alcohol-related programs and services. The senior advisor also interviewed experts in other northern jurisdictions who had been involved in developing alcohol strategies, particularly with those who worked to develop an Indigenous-led strategy in northern Manitoba, a region facing similar challenges to those experienced in the NWT.

### Targeted engagement

#### Liquor legislation review engagement process

Early on in developing the approach to engagement, it was recognized that a parallel engagement process on alcohol, the Liquor Legislation Review (LLR), was taking place. As both the LLR and the Alcohol Strategy were beginning public engagement within a similar timeframe, there were concerns that it would be difficult for community members to distinguish between the two and that they might have the impression that they were being asked to provide feedback twice on the same topic. To address this, the Senior Advisor responsible for the LLR invited the Senior Advisor on Problematic Substance Use to attend the LLR engagement sessions with RCMP and Indigenous and community leaders, to explain the difference between the two processes and capture feedback that fell outside of the scope of the LLR but could be incorporated into the Alcohol Strategy.

#### Addiction recovery experiences survey

The Department of Health and Social Services (DHSS) developed an anonymous public survey to capture the perspectives of individuals with lived and living experience of addictions recovery, identify gaps and barriers to accessing addictions recovery supports, and learn more about what services people are accessing in their recovery journeys. Individuals were invited to participate in the survey via social media, online, and radio advertising, and service providers were also provided with the link for the online version and paper copies of the survey to provide to service users. Results of the survey, which garnered 439 responses in both online and paper formats, were used to produce a report which informed the draft recommendations for an alcohol strategy (Government of the Northwest Territories, 2021b).

#### FOXY/SMASH youth engagement

The DHSS contracted with the organization that runs FOXY (Fostering Open eXpression among Youth) and SMASH (Strength, Masculinities and Sexual Health), programs that provide new ways of talking with youth of all genders about sexual health, sexuality, and relationships across the three northern Canadian territories. They were contracted to do this work due to their expertise in supporting youth to discuss topics that can be challenging, as well as their extensive networks of youth experts and leaders. FOXY/SMASH held nine focus groups with a total of 62 youth between the ages of 13 and 17. The findings from these focus groups were organized thematically into a report that was provided to the DHSS and used to inform the Alcohol Strategy recommendations.

#### Addiction pathways mapping project

The Addiction Medicine and Treatment Working group, created by the senior advisor for the purposes of this project and consisting of addiction service providers from communities throughout the territory, oversaw the development of terms of reference and the selection of a contractor to conduct a review of treatment pathways in order to identify service gaps that exist, gaps in practitioner knowledge and training, and places where individuals may be lost to treatment due to issues with referral pathways and lack of follow-up. The Pathways Mapping Project included a review of territorial government documents, as well as a series of stakeholder engagements consisting of interviews and focus group sessions with a total of 56 participants. DPRA Canada, the successful contractor, drew participants from the DHSS, operations and service provision staff, Indigenous governments and organizations, non-governmental organizations, corrections staff, service users, psychiatrists, and out of territory facilities staff. The report produced included ten observations with accompanying recommendations that were either accepted or modified by members of the working group, and these recommendations (as amended) were used to inform the Alcohol Strategy.

#### Review by advisors and advisory groups

Throughout the process of developing the Alcohol Strategy, there were a number of sessions in which the details of the strategy were presented to key stakeholders for feedback on both the process and content. The Elder who served as Chair of the Minister’s Forum on Community Wellness and Addiction acted in an advisory capacity throughout the process to provide guidance on how to ensure that we were building on this work and following the core principles. The Health and Social Services Indigenous Advisory Body, a group mandated to provide guidance to the DHSS and territorial health authorities on incorporating Indigenous traditions, culture, and healing practices, received regular updates and provided advice at each juncture. The Mental Wellness and Addictions Recovery Advisory Committee, a committee made up of individuals with lived experience to advise the DHSS, received three presentations throughout the development process, so that they could ask questions and provide feedback on the approach to developing the strategy, the recommendations, and the final actions in the Alcohol Strategy.

### Draft recommendations document

The initial recommendations document outlined the preliminary findings of the literature review and engagement process, and included a total of thirty-five recommendations, describing the evidence and rationale for each. The recommendations document included all CAPE policy domains, as well as recommendations associated with reducing alcohol-related harms through prevention, early intervention, and treatment, and recommendations on ways to address key barriers to addressing alcohol-related harms.

### Leadership engagement

The draft recommendations document was circulated for feedback to stakeholders within the Government of the Northwest Territories, and two 3-day virtual meetings were held in March 2022 to present the recommendations, and the supporting evidence, to representatives from Indigenous governments, Indigenous organizations, and community governments. These meetings were attended by representatives from twenty Indigenous governments, Indigenous organizations, and community governments from across the territory. Feedback from participants on each recommendation was compiled into a report, which included the results of a prioritization exercise in which participants were asked to state whether they thought each recommendation was a priority for the territorial government.

### Alcohol strategy

Based on the results of the leadership engagement, an Alcohol Strategy was drafted including a total of fifteen actions, grouped under the themes of communications, policy, prevention, public safety, and treatment. In some cases, similar recommendations were grouped into a single action. For instance, CAPE recommendations about communicating the risks of alcohol use, promoting low-risk drinking guidelines, reducing stigma, and FASD prevention, were combined with other messaging proposed during engagement sessions into an Alcohol Strategy action to create an interdepartmental body to oversee alcohol-related information campaigns. Other recommendations were deemed of low significance by leadership or not applicable within the context of the NWT and were eliminated from the strategy. Finally, some recommendations were eliminated as they were not seen to be the purview of the GNWT. Alcohol labelling has been shown to be a valuable intervention, and there was support for more comprehensive labelling on alcohol products, but given the experience of industry legal challenges when the Yukon piloted a similar approach (Vallance et al., 2020), it was decided that we should support this recommendation at the federal level, but not pursue our own labelling requirements as a territory.

### Validation

**Fig. 2:**
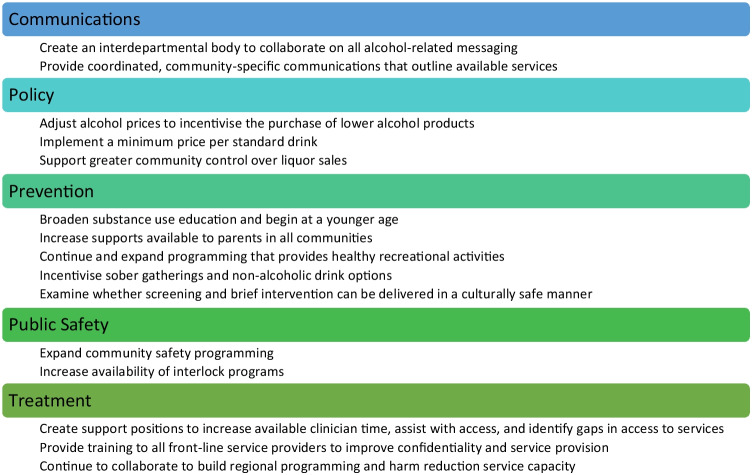
NWT alcohol strategy actions

Community validation was an essential step in the strategy development process due to the diversity that exists between NWT communities in terms of population size, local priorities, road accessibility, availability of alcohol, and cultural practices and beliefs. The draft Alcohol Strategy was circulated to all leaders of Indigenous governments, community governments, and Indigenous organizations and was provided to leaders participating in the NWT Association of Communities Annual General Meeting. The Senior Advisor on Problematic Substance Use attended round table discussions at this meeting to provide an overview of the actions included in the Alcohol Strategy and discuss various elements with community leaders. Presentations were also given at the NWT Council of Leaders meeting, and to the DHSS Indigenous Advisory Body. Feedback was incorporated into the draft strategy and a draft work plan was created and shared with all relevant NWT government departments. Department representatives were asked for their feedback on the strategy and workplan, and departments worked to create consensus around the roles they would take on in implementing each action. A final draft was circulated to departments for approval, and the NWT Alcohol Strategy was tabled in the NWT Legislative Assembly and released publicly on March 20, 2023. Figure 2 summarizes the fifteen actions included in the final Alcohol Strategy.

## Outcomes

### Recommendations already in effect

One reason for the exclusion of CAPE recommendations from the NWT Alcohol Strategy was that some recommendations had already been enacted since the 2019 CAPE project took place. Alcohol pricing and associated government markups were under review in the territory in 2020, with an updated pricing scheme presently under consideration by the Northwest Territories Liquor and Cannabis Commission (NTLCC) and the Department of Finance (Finance). As well, the Motor Vehicles Act in the NWT changed in 2018 to require that all drivers under 22 years of age drive with a BAC of .00 (RSNWT 1988, c M-16, n.d.). Mandatory responsible beverage training, while delayed during COVID-19, is a requirement for all liquor-serving staff and onsite managers of licensed premises, and is now available online.

### Recommendations included in the NWT alcohol strategy

Minimum pricing is a policy lever both recommended in the CAPE report (Vallance et al., 2021) and included in the NWT Alcohol Strategy. This recommendation is supported by evidence that increasing alcohol prices reduces the rates of alcohol dependence and alcohol-related incidents (Stockwell et al., 2006), and that young drinkers and very heavy drinkers, two populations at high risk of alcohol-related harms, are the most price-sensitive (Chaloupka et al., 2002). Aiding in the argument for minimum pricing is the pressure by license holders to reduce restrictions on advertising in the Northwest Territories, which are some of the most stringent in Canada; creating a minimum price will help to ensure that license holders do not have the opportunity to use very low happy-hour drink prices or drink specials, such as one-dollar drinks, to bring in customers, as this also encourages increased alcohol consumption and associated harm (Kuo et al., 2003).

Screening, brief intervention, and referral (SBIR) for alcohol use is also included in the actions in the final Alcohol Strategy but requires further consideration before implementation. While the evidence supports the use of SBIR in clinical and community settings (Vallance et al., 2021), there are considerations around cultural safety that need to be taken into account. The experiences of racism encountered by Indigenous patients within the Canadian healthcare system, including instances where racist stereotypes about substance use have led to the deaths of Indigenous patients (Phillips-Beck et al., 2020), may mean that Indigenous patients may experience an intervention like SBIR as discriminatory, harmful, and/or alienating, further impacting their ability to trust care providers and access effective care. The NWT Alcohol Strategy action includes the need to consider the appropriate setting and service provider when implementing SBIR within the NWT.

### Recommendations adapted for the NWT alcohol strategy

Some recommendations were relevant for the NWT but could not be implemented as written in the CAPE report. The recommendation that the NWT make participation in an ignition interlock program^2^ mandatory for all criminal code convictions for impaired driving is not feasible at this time, due to a lack of mechanics in the NWT who are certified to install this type of device. At present, access to this program is voluntary in the NWT, and is restricted to those who can afford to drive to or have their vehicle transported to a mechanic who can install such a device. The action, as presented in the NWT Alcohol Strategy, is to expand access, beginning with increasing community awareness and gauging community interest in local implementation.

Public reporting of alcohol-related and other social indicators began during the pandemic, to monitor the impacts of COVID-19-related measures on substance use and mental health. While the intent is to continue and expand upon the monitoring and reporting as part of the Alcohol Strategy work, this will be part of the accompanying monitoring and evaluation of each action, as opposed to a stand-alone action in the strategy itself.

### Recommendations not included in the NWT alcohol strategy

The CAPE report recommended it be legislated that health and safety messages be displayed in all on-premises and off-premises establishments, with a variety of messages. Rather than include this in legislation, the NTLCC intends to create this through the licensing process, so that license holders are required to post messaging based on the requirements for their license type. As this change was already planned as part of the NWT Liquor Legislation Review, it was not included in the Alcohol Strategy.

Increasing minimum administrative suspensions for driving under the influence was considered less relevant to the NWT due to the challenges of enforcing these restrictions in northern communities and on the land. Criminology research has shown that certainty of punishment acts as more of a deterrent than severity of punishment, suggesting that increased enforcement initiatives may be more effective than increased penalties (Wright, 2010). Given that increasing penalties may not increase the likelihood of being caught driving under the influence, it was decided that the creation of community safety programs could be more effective. These programs increase the time RCMP have available for highly visible non-selective testing, such as sobriety checkpoints, that are more effective in addressing this issue because they increase public perception of the likelihood of apprehension (Alcohol and Public Policy Group, 2010). Social responsibility messaging will also be used to reduce the social acceptability of operating any motor vehicle while under the influence.

The suggestion of raising the legal drinking age to 21 was considered as part of the development process, but there was not much support for such a change, and youth under 19 reported that they obtained alcohol easily from bootleggers, their parents, or other adults. This suggests that a key rationale for raising the legal drinking age—reducing the availability of alcohol to people under 19 who might obtain alcohol from older friends or siblings—does not apply as readily in the NWT, where bootleggers are common and are unconcerned with the age of their customers. It was determined that addressing bootlegging in communities would be a first step towards reducing youth access to alcohol, rather than raising the legal drinking age.

Restricting outlet density was perceived as a measure that only had the potential to impact the City of Yellowknife, as most communities outside of Yellowknife have few or no licensed premises and one or no liquor stores. If there were to be issues related to outlet density in Yellowknife, it was deemed that this is something that could be acted on at a municipal, rather than territorial, level, as restrictions on outlet density in small communities would likely be difficult to follow given the small population and geographical footprint of most communities. Similarly, the use of mystery shoppers to test whether staff are asking for proof of age was seen as difficult to implement in small communities where everyone knows everyone, as it is likely that any employee in a liquor store or bar would know the approximate age of any community member hired by such a program.

The Government of the Northwest Territories rejected the recommendation that a health and/or public safety–focused department be responsible for the oversight of alcohol retail and regulation due to a perceived conflict of interest, as the same department would then be responsible for both the promotion and provision of alcohol, as well as discouraging alcohol use and treating alcohol use disorders or prosecuting alcohol-related infractions. While liquor stores in the territory are not an entirely government-owned and government-operated monopoly, they currently operate on a consignment model, in which the territory retains control over pricing, product offerings, and maximum hours of operation. While the government monopoly model has been shown to be better than private or hybrid models, it is unclear whether this is true of monopolies over this consignment model, in which the government retains control over many of the parameters known to increase alcohol-related harm in privatized areas.

There was a desire to improve the alcohol warning labels currently in use in the NWT to include other rotating health messages. However, there was a reluctance to include cancer risk messaging on our territorial labels due to the experience of the Yukon territory. In December 2017, the Yukon Government pulled labels indicating cancer risk that were part of a Health Canada–funded study, due to industry threats of legal action (Stockwell et al., 2020). It was determined that, while information about cancer risk should be included in alcohol labelling, this should be pursued at the federal level. As well, information about the number of standard drinks in a container should be included on alcohol labelling; however, due to the wide variety of products and low sales volume relative to Canadian provinces, this is also something that should ideally be required at the federal level and applied by the manufacturer, rather than calculated, produced, and applied by territorial distributors or retailers.

### Recommendations not included in the CAPE report

A policy action that has been applied in Nunavut and was recommended in the NWT Alcohol Strategy is to use a variable product markup based on the alcohol content of a beverage (alcohol by volume, or ABV). In Nunavut, this applies to beer and coolers over 7% ABV, wine over 16% alcohol, and spirits over 30%. At present, markups in the NWT are applied based on product type, regardless of alcohol content. As the variety of products on the market in each category becomes increasingly diverse, with broader ranges of ABV in each product category, the NWT Alcohol Strategy suggested using differential markups based on alcohol content to incentivize the purchase of lower alcohol content products.

At present, messaging on health and safety around alcohol use is siloed into several different departments, with health, harm reduction, social responsibility, and driving under the influence messaging produced and resourced by varying divisions and departments depending on the subject and towards whom it is targeted. Based on discussions with other government departments and agencies, it made sense for these entities to combine efforts, and resources, to create a collaborative body responsible for coordinated messaging that covers all alcohol-related health and safety topics in a strategic manner. This working group was created in May 2023 and continues to meet, and the scope has since been expanded to include all messaging on alcohol and other drugs.

### Actions outside of the policy environment

While the CAPE report is clearly focused on policy frameworks around alcohol and alcohol use, it was clear very early on in the engagement process that an Alcohol Strategy for the NWT would be expected to include improvements to services and new programming designed to prevent alcohol-related harms and support those already struggling with alcohol use disorders. Prevention elements include broadening substance use education; increasing supports for parents; providing more healthy recreational activities for youth, adults, and families; and incentivizing alcohol-free gatherings and non-alcoholic drink options in existing licensed establishments. Harm reduction and treatment initiatives included in the alcohol strategy seek to increase non-police-based community safety programming, creating support positions to help navigate addiction treatment pathways and increase the capacity of clinical staff by providing more training to all front-line staff in confidentiality, substance use, and trauma-informed care. There is also an intent to collaborate with Indigenous governments to build culturally relevant treatment capacity within communities and regions.

## Discussion

The approach used to develop the Alcohol Strategy was able to capture a broad range of perspectives from people living in nearly all NWT communities. Working with an Indigenous Elder with extensive knowledge and experience of substance use and addiction from across the territory allowed us to build on past work, and most importantly, on previous community engagement. It also ensured that we were adding to the body of knowledge in ways that were culturally safe and respectful. Contracting external groups to do some of the engagement activities allowed us improved access to the views of youth, by using an organization that has built broad trust and open communication with this demographic. This approach also improved the feedback we received from service providers, as having an external researcher collecting and analyzing data increased respondent confidentiality and allowed staff to speak more candidly about issues and gaps in the system.

In the context of the Northwest Territories, effective engagement with community members and leadership generally hinges on relationship building and in-person conversations. Unfortunately, the work described in this paper took place almost entirely under strict public health restrictions related to COVID-19. As a result, the in-person meetings we had planned were delayed and ultimately cancelled, switching to a virtual format for nearly all of our stakeholder conversations. This offered fewer opportunities to hear from people who did not actively choose to engage with the online process, or those who did not have the ability to join virtual sessions due to a lack of available technology and restricted internet. In addition to in-person meetings, there are other potential changes that could be made in the future to increase the quality of the input received. Some of the methods used, such as the stakeholder meetings held for the Liquor Legislation Review, or the Addiction Recovery Experiences Survey, may have introduced self-selection bias into the results, as they required an active decision on the part of individuals to participate. As a result, there may be important perspectives that were not captured in the strategy that differ from those of the individuals who actively sought out opportunities to share their views. Techniques to incentivize participation, such as offering compensation to respondents in the form of a gift card or draw entry, could have been used to encourage people who might not otherwise be willing to speak up to share their views and experiences. Some groups of individuals, such as light to moderate drinkers, might not see themselves as being at risk of alcohol-related harm, and therefore might not see the point in participating. Wider distribution of information about the impacts of alcohol and alcohol-related harm on the entire population, as well as the types of policy levers that can be used to reduce alcohol-related harm, may also have raised interest in the topic and elicited more widespread participation.

## Conclusion

The actions outlined in the Alcohol Strategy lay out an approach to reducing alcohol-related harm in the Northwest Territories that considers the views and priorities of stakeholders throughout the Northwest Territories. Continued monitoring and evaluation is needed to assess the effectiveness of the strategy on impacting alcohol use and harm. Plans are to assess and update the strategy in 2028.

## Contributions to knowledge

What does this study add to existing knowledge?

- This paper provides an overview of activities used to assess evidence-based alcohol policy against the unique context and cultures in the Northwest Territories. Jurisdictions seeking to build tailored approaches to addressing alcohol-related harm and other substance use–related issues can build on this approach to considering evidence-based policy approaches against their own local circumstances and needs to build a consensus-based approach.

What are the key implications for public health interventions, practice, or policy?

- This project demonstrated that there is no one-size-fits-all approach to the creation of strategies to reduce alcohol-related harm. It is crucial to examine the evidence and policy best practices within the local context before moving forward with implementation.
- In many cases, policy research is conducted in larger centres and among populations that may not be reflective of rural and remote regions in Canada. The approach outlined in this paper using literature review, multiple engagement methods, and iterative feedback could be applied in various contexts to build community-informed approaches to addressing alcohol-related harm.

## Data Availability

Reports generated from engagement activities, and the final Alcohol Strategy that resulted from this work, are available online. Raw data from these activities are property of the Government of the Northwest Territories and subject to their data access processes.

## Footnotes

1 The Canadian definition of a standard drink is 17.05 mL or 13.45 g of pure alcohol, the equivalent of a 12 oz bottle of 5% beer, a 5 oz glass of 12% wine, or a 1.5 oz shot of 40% alcohol spirits.

2 Ignition interlock programs are programs in which someone installs a device into their vehicle ignition that requires them to blow into a machine and register a 0%/mg blood alcohol content before their vehicle will start. If the individual fails this test, the machine will register it and report failed attempts to the program administrator.
